# Contextualizing Blood‐Based Biomarkers for Dementia Globally

**DOI:** 10.1111/jnc.70443

**Published:** 2026-04-20

**Authors:** Claudia Duran‐Aniotz, Matias Pizarro, Joaquin Migeot, Salomón Salazar‐Londoño, Hernando Santamaría‐García, Sid E. O'Bryant, Agustín Ibanez

**Affiliations:** ^1^ Latin American Brain Health Institute (BrainLat) Universidad Adolfo Ibanez Santiago Chile; ^2^ Global Brain Health Institute (GBHI) Trinity College Dublin (TCD) Dublin Ireland; ^3^ School of Psychology, Center for Social and Cognitive Neuroscience (CSCN) Universidad Adolfo Ibanez Santiago Chile; ^4^ Pontificia Universidad Javeriana Bogotá Colombia; ^5^ Department of Neurology, Alzheimer Center Amsterdam Amsterdam University Medical Center, Vrije Universiteit Amsterdam the Netherlands; ^6^ Neurochemistry Laboratory, Laboratory Medicine Department Amsterdam University Medical Center, Vrije Universiteit Amsterdam the Netherlands; ^7^ Amsterdam Neuroscience Neurodegeneration Amsterdam the Netherlands; ^8^ PhD Program of Neuroscience Pontificia Universidad Javeriana Bogotá Colombia; ^9^ Hospital Universitario San Ignacio Centro de Memoria y Cognición, Intellectus Bogotá Colombia; ^10^ Fundación Santa Fe de Bogotá Bogotá Colombia; ^11^ Institute for Translational Research University of North Texas Health Science Center Fort Worth Texas USA; ^12^ Department of Family Medicine, Texas College of Osteopathic Medicine University of North Texas Health Fort Worth Texas USA; ^13^ Department of Biophysics, School of Medicine Istanbul Medipol University Istanbul Türkiye; ^14^ Barcelonaβeta Brain Research Center (BBRC) Pasqual Maragall Foundation Barcelona Spain; ^15^ Cognitive Neuroscience Center (CNC) Universidad de San Andrés, & CONICET Buenos Aires Argentina

## Abstract

Blood‐based biomarkers (BBMs) are transforming the diagnostic landscape of Alzheimer's disease by enabling scalable, less invasive, and potentially earlier biological characterization. However, most evidence supporting their performance, interpretation, and clinical integration derives from highly selected cohorts in high‐income settings, raising concerns about external validity, threshold transportability, and equitable implementation across diverse populations. In this Opinion, we argue that advancing BBMs from analytical validity to real‐world use requires a shift from biomarker‐centric accuracy toward context‐aware interpretation frameworks that explicitly account for social, environmental, and health system determinants. Using the amyloid, tau, and neurodegeneration (AT(N)) system as a conceptual anchor, we discuss how BBMs should be positioned according to clearly defined contexts of use, including triage, diagnostic support, prognosis, and clinical trial readiness, rather than treated as universal diagnostic substitutes. We examine how social determinants of health, life‐course exposures, and the cumulative exposome interact with comorbidity burden, systemic physiological stress, and health system readiness to shape biomarker distributions, trajectories, and clinical meaning. Evidence from Latin America and other underrepresented settings illustrates how cardiometabolic, vascular, and inflammatory load can modify baseline biomarker levels, challenging the uncritical transfer of cutoffs, reference ranges, and predictive models developed in high‐income settings. We conclude that BBMs hold substantial potential to expand access to biological characterization of Alzheimer's disease, but their responsible adoption depends on aligning biological signals with clinical context, social and environmental conditions, and system capacity. Without this alignment, large‐scale deployment risks misclassification, inequitable access to care, biased trial enrollment, and distorted estimates of disease burden.

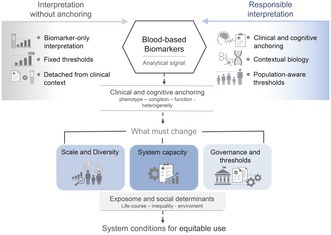

## Graphical abstract

Blood‐based biomarkers are transforming how Alzheimer's disease can be detected and monitored, but their clinical meaning cannot be separated from the contexts in which they are used. This article argues that the value of these biomarkers depends on integrating biological signals with cognitive and functional assessment, life‐course exposures, social and environmental conditions, and health‐system context. The main message is that responsible and equitable implementation of blood‐based biomarkers requires moving beyond fixed thresholds toward interpretation frameworks that are clinically anchored, context‐sensitive, and globally relevant.

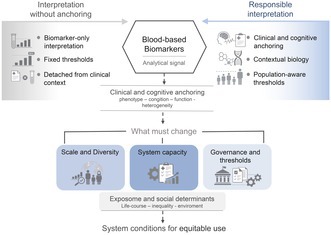

**Abbreviations**
AD: Alzheimer's disease
AT(N): amyloid, tau, and neurodegeneration framework
Aβ42/40: amyloid beta 42/40 ratio
BAG: Brain Age Gap
BBAG: biobehavioral age gap
BBM: blood‐based biomarker
BBMs: blood‐based biomarkers
CSF: cerebrospinal fluid
DTC: direct‐to‐consumer
FINGER: Finnish Geriatric Intervention Study to Prevent Cognitive Impairment and Disability
GFAP: glial fibrillary acidic protein
NfL: neurofilament light chain
NIA‐AA: National Institute on Aging–Alzheimer's Association
PET: positron emission tomography
SDH: social determinants of health
TDP‐43: TAR DNA‐binding protein 43

## 1 Introduction

**FIGURE 1:**
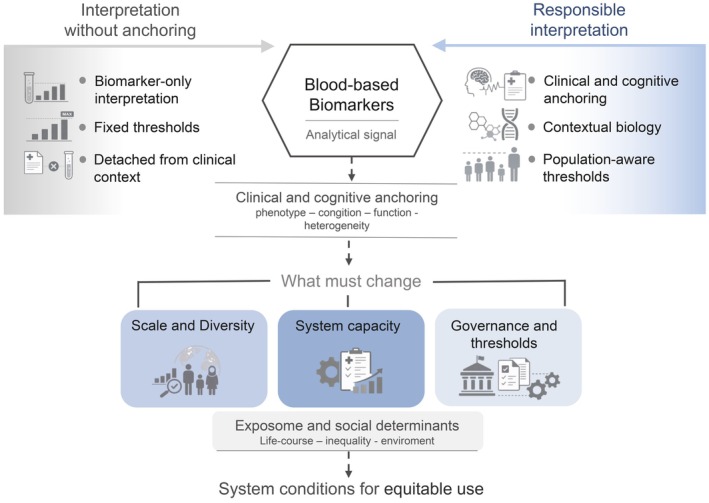
Conceptual framework for the interpretation of blood‐based biomarkers in dementia. Blood‐based biomarkers provide analytically robust signals of underlying biological processes, but their clinical meaning depends on how they are interpreted. When used in isolation, relying on biomarker‐only interpretation and fixed thresholds detached from clinical context, their application may be limited. In contrast, responsible interpretation integrates clinical and cognitive anchoring, contextual biology, and population‐aware thresholds. Moving toward such use requires changes at the system level, including increased scale and diversity of studied populations, strengthened healthcare and laboratory capacity, and appropriate governance of thresholds and implementation pathways.

Blood‐based biomarkers (BBMs) are reshaping how Alzheimer's disease (AD) is detected and understood, but their true potential depends on how they are interpreted and applied across global, diverse, and underrepresented populations. A better understanding of the utility and value of BBMs emerges when they are combined with deep clinical assessment. Their meaning is most interpretable when aligned with multimodal phenotypes, where biological signals can be understood in relation to symptom heterogeneity (Fernández Arias et al. 2025; Ossenkoppele et al. 2025). The growing availability of BBMs has intensified longstanding debates about the relationship between biological abnormality and clinical disease, particularly when biomarker signals are interpreted without sufficient anchoring in cognitive and functional expression (Dubois et al. 2024; Jack et al. 2024). In this context, the clinical value of BBMs cannot be fully realized through biomarker‐centric or threshold‐driven frameworks alone. Interpreting these signals in isolation risks detaching molecular abnormality from its clinical meaning, particularly when biomarkers are applied across diverse and underrepresented populations (Dubois et al. 2024; Jack et al. 2024). A more informative interpretation requires an integrated approach that anchors biological signals to cognitive and functional expression, life‐course exposures, ancestry, genetic and epigenetic predispositions, and health‐system context. Within this framework, BBMs should be understood beyond isolated biological abnormality, incorporating clinical anchoring, exposome‐informed variability, disease burden across clinical stages, and system‐level constraints relevant to low‐ and middle‐income settings (Custodio et al. 2024; Mielke et al. 2024; Parra et al. 2023; Rodda et al. 2025). Although the primary focus of this article is on Alzheimer's disease biomarkers within the AT(N) framework, some markers also discussed reflect broader neurodegenerative or neuroinflammatory processes. These interacting dimensions are summarized in Figure 1.

## 2 Biomarkers Beyond Biology: Integrating Clinical, Cognitive, and Regional/Local/Contextual Anchoring

BBMs, including plasma AT(N) markers such as p‐tau181, p‐tau217, amyloid‐β (Aβ)42/40, neurofilament light chain (NfL), and glial fibrillary acidic protein (GFAP), have reshaped the diagnostic landscape of AD by enabling scalable and minimally invasive measurement of core pathophysiological processes (Grande et al. 2025; Janelidze et al. 2024). These biomarkers allow in vivo assessment of amyloid deposition, tau pathology, neuroaxonal injury, and glial activation, supporting earlier biological characterization across the AD continuum. Within this framework, amyloid‐related markers primarily reflect abnormal Aβ aggregation, phosphorylated tau species reflect amyloid‐associated tau dysregulation and disease stage, NfL captures the intensity of neuroaxonal injury, and GFAP reflects astroglial activation, together providing complementary, nonredundant biological information (Brickman et al. 2021; Jack et al. 2018; Schindler et al. 2024).

BBMs ability to detect amyloid and tau abnormalities, monitor neuroaxonal injury, and track dynamic biological change in neurodegenerative disorders has transformed how those disease stages are identified and tracked (Dhauria et al. 2024; Valletta et al. 2025). Longitudinal studies show that plasma p‐tau217 closely mirrors cortical atrophy, cognitive decline, and neuropsychiatric symptoms, while NfL reflects the rate and severity of neurodegeneration across disease stages and can help distinguish neurodegeneration from other neuropsychiatric changes (Ashton et al. 2022; Mattsson‐Carlgren et al. 2020; Trieu et al. 2025). Contemporary diagnostic frameworks, including those proposed by the NIA‐AA and the International Working Group, conceptualize these biomarkers as indicators of underlying biological processes that require integration with clinical phenotype and cognitive performance to inform diagnosis, staging, and prognosis (Dubois et al. 2024; Jack et al. 2018, 2024).

Most validation studies of BBMs have been conducted in high‐income cohorts from the United States and Europe, limiting the generalizability of proposed thresholds and predictive algorithms (McGlinchey et al. 2024; Parra et al. 2023). The diagnostic and prognostic value of these markers increases substantially when interpreted alongside cognitive and functional measures (Bouteloup et al. 2025; Martino‐Adami et al. 2024). Models that integrate plasma p‐tau217 with domain‐specific cognitive performance consistently outperform biomarker‐only approaches, improving classification accuracy and prognostic estimation across disease stages (Fernández Arias et al. 2025; Ossenkoppele et al. 2025). Recent multicenter work further demonstrates that such integrated models enhance correspondence between biomarker trajectories, cognitive decline, and tau‐PET patterns, reinforcing that BBMs are most informative when embedded within multidimensional clinical frameworks (Gao et al. 2023; Montoliu‐Gaya et al. 2025). Emerging evidence suggests that the performance and interpretation of plasma biomarkers may vary across populations with different demographic, educational, and clinical characteristics, highlighting the importance of validating these markers across diverse settings (Borelli et al. 2025; Caviedes et al. 2026; Custodio et al. 2024).

BBMs interpretation also depends on feasibility and health‐system context (Custodio et al. 2024; Schöll et al. 2024). In many regions, access to cerebrospinal fluid (CSF) analysis or advanced structural and molecular neuroimaging remains limited (Parra et al. 2023). Integrating BBMs with locally validated cognitive and functional tools enhances diagnostic precision (Tideman et al. 2025), improves clinical triage, and increases sensitivity to detect meaningful longitudinal change, particularly in settings with constrained specialist resources.

Clinical heterogeneity adds further complexity. Dementia syndromes with overlapping pathologies, including cerebrovascular disease, limbic‐predominant age‐related TDP‐43 encephalopathy, and frontotemporal lobar degeneration, can produce biomarker profiles that partially resemble AD patterns (Gómez‐Tortosa et al. 2025; Liampas et al. 2024). Importantly, many of these conditions currently lack well‐established BBMs, and mixed pathologies are common in clinical populations. Thus, individuals with positive AD biomarkers may have additional contributing pathologies that require consideration in diagnosis and management (Tosun et al. 2024; Frisoni et al. 2025). The prevalence and clinical expression of non‐Alzheimer's dementias also vary substantially across regions and populations, reflecting differences in genetic background, comorbidity burden, and environmental exposures (Cano‐Gutiérrez et al. 2025; Duran‐Aniotz et al. 2021). These overlaps underscore that biomarker abnormalities are not disease‐specific in isolation and require interpretive models capable of distinguishing primary neurodegenerative processes from vascular, inflammatory, or syndrome‐specific contributions, particularly when BBMs are applied outside Global North cohorts (McGlinchey et al. 2024; Schindler et al. 2024).

Embedding BBMs within clinical workflows therefore has implications that extend beyond diagnosis alone. Integrating biomarker data with cognitive and functional assessment supports earlier detection, more equitable referral pathways, and regionally grounded diagnostic strategies that better reflect real‐world patient diversity. We argue for positioning BBMs not as isolated tests, but as components of clinically anchored and context‐responsive models of brain health assessment, aligned with emerging implementation frameworks. The following section extends this view by examining how biomarker variability reflects the cumulative influence of environmental exposures and social determinants of health (SDH) across the life course.

## 3 Understanding Biomarker Variability Through the Exposome and SDH

Dementia biology is shaped by cumulative life‐course exposures, including cardiometabolic burden, infections, educational disparities, psychosocial adversity, and socioeconomic inequality (Da Ros et al. 2025; Ibanez et al. 2024; van der Ende et al. 2019). These exposures converge on shared physiological burden involving systemic inflammation, vascular dysfunction, metabolic strain, and neuroendocrine disruption (Furman et al. 2019; Pantell et al. 2024). Such mechanisms influence both the risk of neurodegeneration and the trajectories captured by BBMs (O'Bryant et al. 2023). BBMs, including GFAP, NfL, and Aβ42/40, may partially reflect the biological consequences of chronic stress exposure, vascular compromise, or pro‐inflammatory activation, and in some contexts may indicate systemic or stress‐related burden rather than preclinical AD pathology (Furman et al. 2019; Pantell et al. 2024). Large‐scale analyses show that individuals exposed to early adversity, poor educational quality, or sustained socioeconomic strain exhibit altered proteomic signatures enriched in inflammatory, oxidative, and vascular pathways, patterns that interact directly with amyloid biology, tau phosphorylation, and neuroaxonal injury (Da Ros et al. 2025; Palmqvist et al. 2024; van der Ende et al. 2019), highlighting how biological abnormalities may reflect regulated or dysregulated responses to structural determinants rather than purely primary AD mechanisms.

Emerging work quantifies how environmental and social exposures become biologically embedded (Ibanez et al. 2024). Brain Age Gap (BAG) studies reveal that adverse exposures accelerate the difference between chronological and predicted brain age, indicating accelerated biological aging (Hernandez et al. 2025). Extending this concept, biobehavioral age gap (BBAG) analyses in more than 160 000 individuals demonstrate that macro‐level exposome dimensions, including air pollution, income inequality, gender representation, and political participation, are robust predictors of accelerated biological aging (Hernandez et al. 2025). More recently, the compound effects of dozens of physical and social exposomal factors have shown substantially larger effects in terms of accelerated brain aging, comparable to or larger that those observed with dementia (Hernandez et al. 2025; Legaz et al. 2025; Migeot et al. 2025). These studies illustrate how upstream social and environmental forces materially reshape biomarker expression and brain vulnerability across populations.

Variation in biomarker profiles also reflects differences in vulnerability and resilience shaped by SDH (Borelli et al. 2025). Access to healthcare, occupational strain, and food insecurity interact with biological mechanisms to shape aging and dementia phenotypes (Krishnamurthy et al. 2025; Migeot et al. 2025). Chronic exposure to adversity can lead to sustained stress overload, which has been linked with white‐matter hyperintensities and ventricular enlargement (Altschuler et al. 2025; Palix et al. 2025), demonstrating how stress‐related physiological burden affects brain structure in ways that overlap with pathological neurodegeneration. Structural inequities in access to care, diagnostic delays, untreated comorbidities, and fragmented health systems further distort biomarker expression by elevating markers that reflect systemic disadvantage rather than AD‐specific pathology (Frisoni et al. 2025; McGlinchey et al. 2024). These combined inequities interact with vascular, metabolic, and inflammatory pathways, reinforcing the importance of contextualized frameworks for interpreting BBMs in diverse populations.

Overall, these findings indicate that biomarker interpretation must extend beyond molecular pathology to incorporate life‐course exposures, regional socioeconomic conditions, and clinical heterogeneity. When the biological embedding of environmental and social adversity is not accounted for, variability in BBMs may be misattributed to primary neurodegenerative processes, potentially leading to under‐ or overdiagnosis and misclassification of individuals whose biomarker profiles predominantly reflect vascular, inflammatory, or stress‐related burden. These profiles may derive from proteomic and other molecular signatures reflecting vascular dysfunction, inflammatory activation, metabolic strain, and stress‐related biological burden, which may influence BBM levels independently of, or in interaction with, AD‐related pathology (Hampel et al. 2023; Valletta et al. 2024; Inamdar et al. 2025). Such misinterpretation can distort estimates of disease risk and progression and has downstream implications for clinical trials and population‐based studies, where uncontextualized biomarker thresholds may reduce precision, bias participant selection, and limit the validity of BBMs as outcome measures in diverse populations. Addressing these sources of variability is therefore essential to ensure that BBMs capture disease biology rather than the unequal distribution of environmental and social risk.

## 4 From Diagnosis to Disease Burden

Interpreting BBMs requires careful consideration of disease stage (Ashton et al. 2022; Jack et al. 2018; Schindler et al. 2024). Diagnostic thresholds commonly used to classify individuals as biomarker‐positive or biomarker‐negative have largely been derived from selected, high‐income, and relatively homogeneous cohorts (Karikari 2022; Mielke and Fowler 2024). Applying such thresholds to diverse populations without adjustment risks misclassification, inaccurate estimation of disease prevalence, and inequitable access to care, because identical biomarker values may reflect different underlying biological and clinical states depending on baseline comorbidity burden, cumulative life‐course exposures, background prevalence of AD pathology, and health‐system diagnostic pathways. These limitations become particularly salient when diagnostic cutoffs are applied across health systems with different background prevalence, comorbidity profiles, and diagnostic pathways, where identical values can shift the balance of false positives and false negatives in clinically meaningful ways (Jack et al. 2024; Schindler et al. 2024). BBM interpretation must therefore account for inter‐individual variability informed by ancestry, comorbidity, social context, and cumulative stress burden.

In settings where access to neuroimaging modalities such as MRI or PET remains limited, BBMs offer a scalable alternative for monitoring disease burden and informing clinical decisions, as they require less specialized infrastructure, can be deployed across different levels of care, and allow lower‐cost and higher‐frequency assessment than advanced imaging (Barthélemy et al. 2024; Prufer et al. 2025). Their feasibility for repeated sampling enables longitudinal assessment in low‐resource environments where advanced diagnostics may be scarce. When applied across the diagnosis‐to‐prognosis continuum, BBMs can support risk stratification, help identify individuals at higher likelihood of progression, and guide allocation of limited healthcare resources (Hampel et al. 2023; Mielke et al. 2024). However, these benefits depend on context‐sensitive interpretation frameworks capable of distinguishing AD‐related pathological change from biomarker elevations influenced by vascular, metabolic, or inflammatory conditions that may interact with AD pathology. Even PET and CSF demonstrate variability and discordance across settings, particularly in populations with lower AD prevalence or greater etiologic heterogeneity.

Population‐level data further indicate that the prognostic value of BBMs varies by disease stage. In community‐based cohorts, p‐tau217, NfL, and GFAP demonstrate strong predictive utility for identifying individuals with mild cognitive impairment at higher risk of progression but show limited ability to forecast decline among cognitively unimpaired individuals (Valletta et al. 2025). These findings support a shift toward probabilistic, risk‐based BBMs strategies that incorporate continuous biomarker distributions, comorbidity‐adjusted thresholds, and multimodal integration rather than rigid binary classification. Beyond diagnosis and prognosis, BBMs can also function as surrogate endpoints in clinical trials, capturing pharmacodynamic responses that may precede measurable clinical benefit (Hansson et al. 2023; Mattsson et al. 2019; Oosthoek et al. 2024). To extend this role beyond highly selected trials, evidence must expand to larger and more representative participant samples spanning research cohorts and real‐world care pathways in underrepresented regions (Galvin et al. 2024; Kjaergaard et al. 2025; Schöll et al. 2024).

## 5 Biomarkers as Tools for Monitoring Interventions and Real‐World Implementation

Finally, the translation of BBMs into real‐world practice depends on implementation frameworks that bridge research, clinical capacity, and governance. Despite rapid scientific progress, the clinical adoption of BBMs remains uneven. Surveys reveal that many clinicians lack confidence in their use, citing limited training, insufficient interpretation tools, and lack of validation across diverse populations (Deverka et al. 2025; Palmqvist et al. 2024; Rodda et al. 2025). In low‐ and middle‐income regions, implementation requires embedding BBMs into existing healthcare infrastructures, expanding workforce capacity, and developing locally informed protocols for triage and diagnostic referral (Erickson et al. 2025; Korologou‐Linden et al. 2024). Without such coordination, BBMs risk remaining research tools rather than supporting real‐world diagnostic decision‐making. Expanding BBMs' use across both research cohorts and routine clinical care is therefore critical to generate locally valid performance estimates, reference ranges, and implementation‐ready algorithms that reflect real‐world heterogeneity and disease prevalence (Frisoni et al. 2025; Jack et al. 2024).

Community‐based and regional research initiatives demonstrate that coordinated BBMs deployment is feasible within structured study settings, even in resource‐constrained environments. ReDLat illustrates how BBMs use can be operationalized within multicenter intervention studies through regional laboratory hubs, harmonized analytic workflows, and culturally adapted training frameworks (Kivipelto et al. 2020; Udeh‐Momoh et al. 2024).

While these initiatives do not represent national‐scale health system implementation or routine primary care deployment, they provide important proof‐of‐concept evidence for the logistical, analytic, and training components required to support BBMs use across diverse regions. At the same time, implementation depends on addressing practical barriers including laboratory equipment, assay availability, sample logistics, quality control, and sustained training for clinicians and laboratory personnel, which determine whether BBMs can move from proof of concept to routine use (Aye et al. 2024; Hansson et al. 2023; Zeng et al. 2024).

At the health‐system level, current clinical practice guidelines recommend using BBMs primarily as triage tools for symptomatic individuals, with CSF or PET confirmation when available (Palmqvist et al. 2025). Implementation studies emphasize that simplified reporting formats, locally adjusted thresholds, and comorbidity‐informed interpretation models are essential to minimize misclassification in populations with high clinical and environmental heterogeneity (Frisoni et al. 2025). As health systems scale BBMs use, the development of robust quality‐control procedures, laboratory certification standards, and interoperability across clinical settings becomes increasingly important (Daly 2025). These governance and quality frameworks are also foundational for building clinically usable platforms for trials, where BBMs support participant identification, stratification, monitoring, and equitable access to emerging disease‐modifying therapies across regions (Frisoni et al. 2025; Pascoal et al. 2024).

A growing concern is the rapid expansion of direct‐to‐consumer (DTC) biomarker testing. Unregulated DTC access raises psychological, ethical, and clinical risks by providing biomarker results without appropriate counseling, diagnostic context, or pathways for follow‐up care (Grill 2026). At a systems level, widespread DTC testing may strain health services as individuals seek confirmatory evaluation, particularly in settings where access to neuroimaging or specialist review is already limited. These developments underscore the need for global and national governance frameworks that regulate assay quality, reporting standards, and consumer protection (Daly 2025).

As disease‐modifying therapies emerge, BBMs are poised to play an increasingly central role in monitoring pharmacodynamic responses and treatment‐related biological change, as well as in identifying individuals most likely to benefit from therapeutic or preventive interventions (Angioni et al. 2022; Korologou‐Linden et al. 2024). BBMs may also expand access to clinical trials by enabling scalable prescreening and longitudinal monitoring, reducing structural barriers that have historically excluded underrepresented populations. To fully realize this potential, trial‐ready BBMs platforms require alignment between laboratory capacity, clinical workflows, and regulatory oversight, particularly in underrepresented regions where access to trials has been historically limited (Matton et al. 2025; Teunissen et al. 2022).

Beyond pharmacological interventions, BBMs support the design and evaluation of multimodal prevention strategies. By identifying individuals at elevated biological risk, BBMs can guide the selection of targeted lifestyle interventions and enable tracking of biological change alongside cognitive or functional outcomes, as demonstrated in multidomain programs such as FINGER (Crivelli et al. 2023; Sala et al. 2025; Udeh‐Momoh et al. 2025). Across clinical, preventive, and population‐health applications, implementation requires sustained investment in laboratory capacity, workforce training, and regulatory oversight to ensure that BBM deployment is both safe and equitable. Collectively, these considerations position BBMs as core components of a broader translational ecosystem. Their successful integration into real‐world practice will depend on aligning scientific innovation with clinical workflows, health‐system capacity, and appropriate governance frameworks.

## 6 Future Perspectives and Proposals

Moving beyond biomarker‐centric thresholds is essential to avoid biased diagnosis and suboptimal clinical decision‐making, particularly when cutoffs and algorithms are transported from predominantly Global North cohorts to diverse, underrepresented populations. To move toward globally valid and clinically meaningful BBM use, we propose a contextualized interpretation framework linking biomarker signals to whole‐body biology, population diversity, and real‐world exposures (Hernandez et al. 2025; Ibáñez et al. 2025).

First, BBMs should be interpreted in relation to a multimodal biological context, including neuroimaging, cardiovascular and metabolic profiles, and multi‐omics layers. Integrating genomics, epigenomics, proteomics, and other omics readouts can help distinguish AD‐specific molecular signatures from broader processes such as vascular injury, inflammation, metabolic strain, or accelerated biological aging. This approach is especially important in populations with different baseline comorbidity burdens and in settings where mixed etiologies are common. In parallel, ancestry‐aware modeling is essential: genetic architecture, population stratification, and ancestry‐related variation may influence both baseline BBMs distributions and their diagnostic meaning. Similarly, BBM interpretation should also consider familial or genetic forms of neurodegeneration, where biomarker trajectories and timing may differ from sporadic disease (Aguirre‐Acevedo et al. 2016; Bateman et al. 2012; Lopera et al. 1997).

Second, BBMs variability must be understood through the lens of the exposome and SDH (Hernandez et al. 2025; Ibanez et al. 2024). Across individual and population levels, cumulative exposures shape systemic physiology in ways that modulate BBM levels and their clinical utility (Furman et al. 2019; Krishnamurthy et al. 2025; O'Bryant et al. 2023). At the macro level, global exposomes social, environmental, physical, and cultural, may shift biomarker distributions and alter the balance of false positives and false negatives when fixed thresholds are applied across settings. Without accounting for these exposures, biomarker abnormalities risk being misattributed to primary AD biology, leading to misclassification and inequities in care and research.

Third, improving BBMs interpretability requires strengthening deep phenotyping. Biomarker signals become clinically actionable when anchored to fine‐grained characterization of cognition, function, neuropsychiatric symptoms, and real‐world performance. This is particularly important in individuals with discordant profiles, where interpretation depends on phenotype–biology coupling rather than biomarker status alone (Ibáñez et al. 2025).

Taken together, these priorities define a roadmap for next‐generation BBM frameworks that are more interpretable, equitable, and clinically useful.

## 7 Conclusions: A Global Research Roadmap for Contextualized and Equitable Biomarker Science

BBMs have the potential to transform dementia care by enabling scalable detection of neuropathological processes, improving prognostic precision, and supporting more equitable access to diagnosis and interventions. Yet their impact will depend on developing interpretive frameworks that move beyond models derived from high‐income settings and toward approaches that incorporate clinical expression, population diversity, and contextual complexity. Anchoring BBMs to multimodal phenotypes, including cognitive, functional, and neuroimaging assessments, and integrating genetic and exposome diversity into their interpretation within a whole‐body health framework (Ibáñez et al. 2025), may help characterize underlying pathological processes in a more personalized manner. Without such context‐aware interpretation, biomarker‐based models risk reinforcing existing inequities by transferring thresholds and assumptions that do not reflect the biological and clinical heterogeneity of global populations (Frisoni et al. 2025; McGlinchey et al. 2024).

Recent progress in establishing population‐specific reference ranges, including work from Brazil and studies of Latin American individuals living in the United States, demonstrates the feasibility and scientific value of tailoring biomarker thresholds to regional and ancestry‐related variation (Santos et al. 2025; Varela‐Vidales et al. 2025; Caviedes et al. 2026). These efforts highlight the need for globally normative and precision frameworks that recognize diversity as a cornerstone of biomarker science rather than a deviation from the standard (Ibáñez et al. 2025). Expanding the number and representativeness of individuals with available BBM data across underrepresented regions is therefore critical to generate adequate reference ranges and situated models of risk and progression (Griswold et al. 2025; Mielke and Fowler 2024).

Scaling BBMs across diverse health systems represents a major structural challenge that extends beyond scientific readiness and requires sustained public investment. Achieving this scale will depend on long‐term governmental commitment to laboratory infrastructure, biobanking capacity, and regional manufacturing capabilities, alongside open‐access analytic protocols and cost‐effectiveness evaluations. Implementation guidelines emphasize the need for regulatory and governance frameworks to ensure assay quality, protect individuals from misuse of biomarker information, and support integration into clinical pathways (Daly 2025; Grill 2026). These frameworks are also essential for aligning BBM deployment with clinical trials and prevention programs, where scalable screening, longitudinal monitoring, and equitable access to innovation depend on health‐system readiness (Frisoni et al. 2025; Hansson et al. 2023; Pascoal et al. 2024).

Thus, the future of BBM research lies not only in integrating molecular discovery with real‐world heterogeneity and clinical relevance, but also in confronting the structural and systemic conditions required for sustainable implementation. Equity, contextual understanding, and population specificity must be placed at the core of biomarker science, particularly in regions that have been historically underrepresented and under‐resourced. Within this framework, BBMs are not standalone solutions, but tools whose capacity to reshape dementia diagnosis, monitoring, and prevention ultimately depends on sustained investment, health‐system readiness, and responsible governance.

## Author Contributions

**Salomón Salazar‐Londoño:** writing – review and editing, investigation. **Matias Pizarro:** investigation, writing – original draft, writing – review and editing, visualization. **Claudia Duran‐Aniotz:** conceptualization, investigation, writing – original draft, writing – review and editing, supervision, funding acquisition, resources, project administration. **Agustín Ibanez:** conceptualization, writing – original draft, writing – review and editing, supervision, funding acquisition, resources, project administration. **Joaquin Migeot:** investigation, writing – original draft, visualization, writing – review and editing. **Hernando Santamaría‐García:** writing – review and editing, investigation. **Sid E. O'Bryant:** writing – review and editing.

## Funding

C.D.A. is supported by ANID/FONDECYT Regular (1210622 and 1250091), ANID/NAM22I0007, ANID/PIA/ANILLOS ACT210096, Alzheimer's Association (AARGD‐24‐1310017), ANID/FOVI240065, ANID/Proyecto Exploracion 13240170. M.P. is supported by the National Agency for Research and Development (ANID)/Scholarship Program 2024/National Doctorate 21241636. H.S.‐G. is funded by NIH D43 Fogarty (Research Training for Equity in Alzheimer's Disease and Brain Health in Colombia, number: 1D43TW012455‐01A1), NIH R01 (Social epigenetics of Alzheimer's disease and related dementias in Latin American countries, number: 1R01AG082056‐01A1), Global Brain Health Institute and Alzheimer Association (“Brain health in individuals with exposition to high violence in Colombia”, number: GBHI ALZ UK‐23‐971135). A.I. is supported by grants from the Multi‐partner consortium to expand dementia research in Latin America [ReDLat2, supported by Fogarty International Center (FIC), National Institutes of Health, National Institutes of Aging (R01 AG057234, R01 AG075775, R01 AG21051, R01 AG083799, CARDS‐NIH, R01 AG057234), Alzheimer's Association (SG‐20‐725 707), Rainwater Charitable Foundation—The Bluefield project to cure FTD, and Global Brain Health Institute], ANID/FONDECYT Regular (1250091, 1210176, and 1220995); ANID/PIA/ANILLOS ACT210096; JPI JPND‐Care, DISCeRN 2025—Health and Social Care Research with a Focus on the Moderate and Late Stages of Neurodegenerative Diseases; FONDEF ID20I10152, and ANID/FONDAP 15150012; Wellcome Trust award for BRAIN‐CLIMA: Investigating the Combined Impact of Heat and Air Pollution on Blood–Brain Barrier Integrity and Brain Aging in Latin America (335293/Z/25/Z), Wellcome Leap CARE Program (grant number: CARE‐2025‐0883490149) for the project “Advancing Female‐Specific Predictive Models and Risk Assessment Tools for Alzheimer's Disease in the US and Latin America,” and the CliCBrain (Horizon ID: 101236426; https://doi.org/10.3030/101236426, Marie Skłodowska‐Curie Actions—MSCA).

## Conflicts of Interest

The authors declare no conflicts of interest.

## Data Availability

Data sharing is not applicable to this article because no new data were created or analyzed in this study.
